# Biomechanical Comparison of Self-Compressing Screws and Cortical Screw Inserted with Lag Fashion in Canine Cadaveric Humeral Condylar Fracture Model

**DOI:** 10.3390/vetsci12010072

**Published:** 2025-01-20

**Authors:** Jun-sik Cho, Jung Moon Kim, Youn-woo Choo, Jooyoung Kim, Sorin Kim, Hwi-yool Kim

**Affiliations:** Department of Veterinary Medicine, Graduate School of Konkuk University, Seoul 05029, Republic of Korea; junsik10@gmail.com (J.-s.C.); kimjungm418@gmail.com (J.M.K.); beobjamu@gmail.com (Y.-w.C.); piggy_0818@hanmail.net (J.K.); kimjx3447@naver.com (S.K.)

**Keywords:** humeral condylar fracture, biomechanical comparison, canine, partially threaded cannulated screw, fully threaded headless cannulated screw

## Abstract

This study compares the compression force of cortical screws used in lag fashion with partially threaded cannulated screws and fully threaded headless cannulated screws as fixation methods for humeral condylar fractures in dogs. Cadavers of eleven dogs weighing an average of 10.99 ± 2.51 kg (6.1–14.4 kg) were used. The humeri were subjected to simulated fracture by performing an osteotomy at the trochlea of humerus and classified into three groups: Group 1 applied a 3.0 mm cortical screw applied in a lag fashion, Group 2 applied a 3.0 mm partially threaded cannulated screw, and Group 3 applied a 3.5 mm fully threaded headless cannulated screw. The samples were then placed in a material testing machine, and a compression force was applied vertically to the lateral condyle until failure. There were statistically significant differences in failure load between the groups (*p* = 0.009). The maximum failure load in Group 3 was significantly higher than in Group 2 (*p* = 0.014), while there were no statistically significant differences between Group 1 and Group 2) or between Group 1 and Group 3. Partially threaded cannulated screws and fully threaded headless cannulated screws can be alternatives to traditional stabilization methods, offering simpler procedures and additional advantages.

## Simple Summary

This study aimed to improve the treatment of broken humeral condyles in dogs by comparing three different types of screws used to hold the fractured bones together. We used eleven dog cadavers and divided them into three groups. Each group received a different type of screw for bone repair: a cortical screw applied in a lag fashion, a partially threaded cannulated screw, and a fully threaded cannulated headless screw. Both cannulated screws have a self-compressing ability with simpler procedures. There was no significant difference between the cortical screw and the other two types in biomechanical compression tests. This research may lead to better surgical options for dogs with elbow fractures, resulting in quicker procedures with stability.

## 1. Introduction

Fractures of the humeral condyle are common in the dog, accounting for 41% of fractures in a survey of 107 humeral fractures. The lateral side is fractured more commonly because of anatomic and biomechanical differences, accounting for 34% to 67% of fractures of the humeral condyle and for 37% of all fractures of the distal humerus [1]. These types of fractures are generally associated with moderate trauma and are most seen in animals aged three to four months [2]. Cocker Spaniels are a breed with a higher incidence of condylar fractures, as these fractures often occur alongside incomplete ossification of the humeral condyle in this breed [3].

A lateral condylar fracture consists of a fracture through the narrow midportion of the condyle, immediately medial to the line of the radial head, along with a separate fracture through the lateral metaphyseal ramus. Therefore, effective fracture reduction requires anatomical alignment of both fractures, with particular emphasis on the articular surface of the condyle, to achieve maximal restoration of joint function. Malalignment or insufficient fixation can lead to nonunion, potentially resulting in complete loss of elbow function and necessitating elbow arthrodesis [4].

Transcondylar screws inserted in lag fashion are commonly employed to achieve interfragmentary compression and promote primary bone healing without forming callus on the articular surface. However, inserting screws in lag fashion involves a multi-step procedure that can be both technically challenging and time-consuming [3]. Furthermore, soft tissue impingement resulting from screw head protrusion may lead to long-term discomfort [5].

Various studies of different screws for humeral condylar fractures have been documented. Vida, J.T., et al. (2005) compared partially threaded cannulated screws and partially threaded cancellous screws, and Gonsalves, M.N. et al. (2016) compared partially threaded headless screws and cortical screws [3,6].

A partially threaded cannulated screw (PTCS) has a smooth shaft and the threaded part. The former serves as a gliding hole, and the latter holds the far bone fragment, respectively. As the head of the screw is tightened at the near cortex, interfragmentary compression is created like a lag screw [7].

A fully threaded headless cannulated screw (FTHCS) is designed as a conical shape and a cannulated structure without a head, featuring a shaft that is entirely threaded with a cancellous configuration and a variable stepped thread pitch. The screw’s diameter gradually increases from the tip to the base, while the thread pitch correspondingly becomes finer. Each turn of the thread creates compression because the differences in thread pitch result in slower insertion speeds of the near fragment. Therefore, as the screw is driven deeper into the bone, interfragmentary compression is achieved [8]. In human medicine, FTHCSs are an emerging minimally invasive surgical method, precisely guided by wire and avoiding damage to surrounding tissues [9]. Recently, FTHCSs were used in femoral neck fractures of dogs [8].

To the authors’ best knowledge, there is no study to compare PTCSs and FTHCSs with cortical screws in canine humerus condylar fractures. This study hypothesizes that self-compression screws are as stable as cortical screws inserted using a lag technique and aims to evaluate the stability of self-compression screws compared to cortical screws for treating humeral condylar fractures in dogs.

## 2. Materials and Methods

### 2.1. Cadaver Preparation

Twenty-one forelimbs were obtained from eleven dogs euthanized for reasons unrelated to this study (Incheon Veterinary Medical Association, Incheon, Republic of Korea). The dogs consisted of two Beagles and nine mixed breeds, with an average body weight of 10.99 ± 2.51 kg (6.1–14.4 kg). Five female dogs and six male dogs were included, and the condyle diameters at their narrowest parts ranged from 9 mm to 14 mm.

All cadaveric forelimbs underwent orthopedic examination and radiography to identify bone deformities or any other orthopedic diseases. All the samples were skeletally matured and none of the samples showed abnormalities.

**Figure 1:**
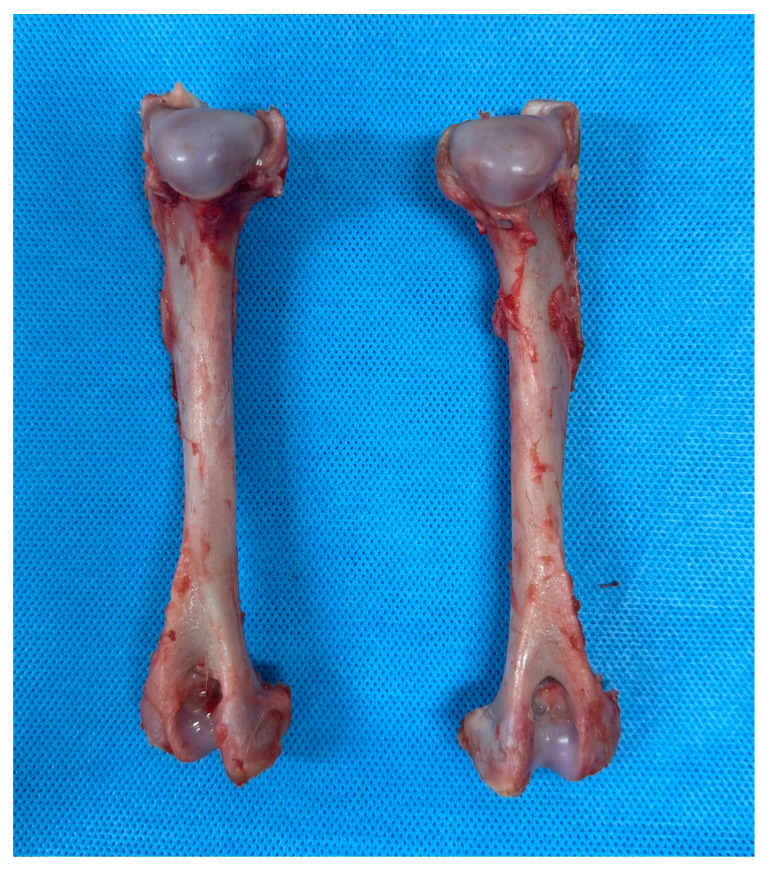
A photograph of cadaver sample of humeri. The humeri were obtained from canine cadavers. No bone deformities or other orthopedic diseases were detected, ensuring that normal bones were included in the study. The condyle diameters at their narrowest parts ranged from 9 to 14 mm.

Disarticulation of the scapulohumeral and humeroulnar joints was performed. Soft tissues were removed from the humerus (Figure 1). The specimens were wrapped in gauze soaked in 0.9% normal saline solution and stored at −80 °C until the experiment. The specimens were thawed at room temperature 24 h before the experiment.

### 2.2. Implant Application

A condylar fracture was simulated by osteotomies in the narrowest part of the condyle in canine cadaveric humeri, as previously described [10]. The lateral fragments were stabilized with each screw. To concentrate the stress on the lateral condyle, bone fragments were removed by performing a 10 mm supracondylar crest osteotomy. To facilitate anchoring of the sample, the proximal part of the humerus was cut at the level of the distal aspect of the deltoid tuberosity using an oscillating saw.

Eleven samples were randomly divided into 3 groups. Groups were classified as follows: 3.0 mm screw with lag fashion (Group 1), 3.0 mm PTCS (Group 2), and 3.5 mm FTHCS (Group 3).

#### 2.2.1. 3.0 mm Cortical Screw with Lag Fashion (Group 1)

**Figure 2:**
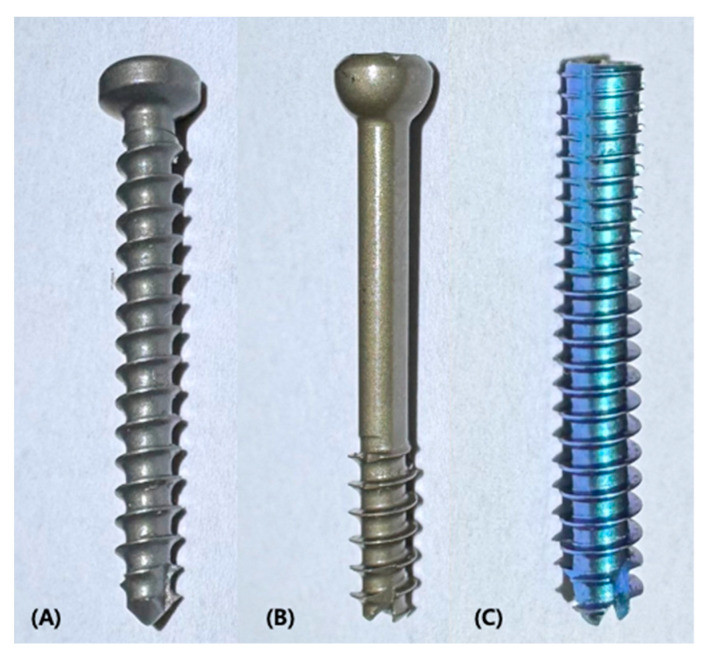
Images of screws used for each group. All screws were manufactured from Arthrex Inc. and were made of titanium. Screws were inserted into the humeral condyle from lateral to medial until adequate compression was achieved. (**A**) A 3.0 mm self-tapping cortical screw (LPS screw Titanium; Arthrex Inc.) was used for Group 1. (**B**) A 3.0 mm PTCS (QuickFix screw; Arthrex Inc.) was used for Group 2. (**C**) A 3.5 mm FTHCS (Cannulated Headless Mini Compression FT screw; Arthrex Inc.) was used for Group 3.

**Figure 3:**
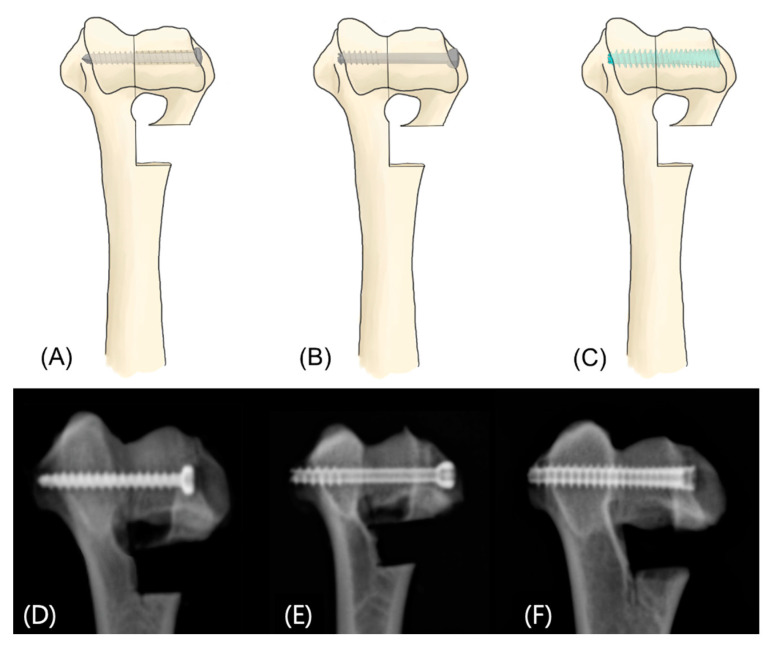
Illustrations and radiographic images of the 3 fixation methods of simulated humeral condylar fracture. Simulated humeral condylar fractures were fixed using each respective method and confirmed with radiography. (**A**,**D**) In Group 1, a 3.0 mm cortical screw was inserted in a lag fashion. (**B**,**E**) In Group 2, a 3.0 mm PTCS was used. (**C**,**F**) In Group 3, a 3.5 mm FTHCS was inserted.

In Group 1, a 3.0 mm self-tapping cortical screw (LPS screw Titanium; Arthrex Inc., Naples, FL, USA) was inserted in a lag fashion according to the standard AO/ASIF technique (Figure 2A). A 1.1 mm guide wire was placed at the screw entry point previously described by Barnes et al. (2014) [11]. Pilot holes were drilled using a 2.0 mm cannulated drill bit (Arthrex Inc.), followed by gliding holes drilled with a 3.0 mm cannulated drill bit (Arthrex Inc.) in the lateral bone fragment. After removing the K-wire, the screws were inserted in a lag fashion and tightened until interfragmentary compression was achieved (Figure 3A,D).

#### 2.2.2. 3.0 mm Partially Threaded Cannulated Screw (Group 2)

In Group 2, a 3.0 mm PTCS (QuickFix screw; Arthrex Inc.) was used (Figure 2B). Using the manufacturer-recommended 3.0 mm cannulated screw system guide as previously described by Kim S. et al. (2024), the PTCS was fixed. A 1.1 mm guide wire was placed at the same entry point as in the Group 1, and a 3.0 mm PTCS was inserted over the guide wire and tightened until interfragmentary compression was achieved (Figure 3B,E). When proper screw fixation was achieved, the guide wire was removed.

#### 2.2.3. 3.5 mm Fully Threaded Cannulated Headless Screw (Group 3)

In Group 3, a 3.5 mm FTHCS (Cannulated Headless Mini Compression FT screw; Arthrex Inc.) was used (Figure 2C). Using the manufacturer-recommended 3.5 mm cannulated screw system as previously described by Kim S. et al. (2024), the FTHCS was fixed. A 1.1 mm guide wire was placed at the same entry point as in the Group 1, and a 3.5 mm FTHCS was inserted over the guide wire and tightened until interfragmentary compression was achieved (Figure 3C,F). When proper screw fixation was achieved, the guide wire was removed.

### 2.3. Biomechanical Testing

**Figure 4:**
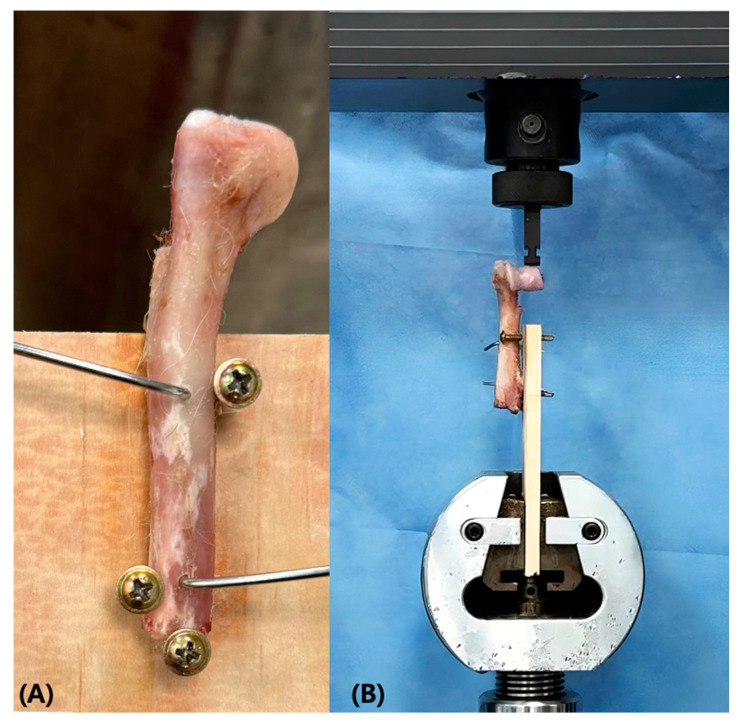
A photograph of biomechanical compression test. (**A**) The sample was fixed to a wooden board. (**B**) After fixation, sample was placed in compression jig to press the highest point of the lateral condyle.

The lateral sides of specimens were fixed vertically to wooden boards positioned parallel to the long axis of the humerus. Two 2.0 mm pins were inserted through the humeral shafts into the wooden boards (Figure 4A). Additionally, two screws were applied on each side of the humeral body to prevent the samples from rotating and dislodging from the boards (Figure 4B). The specimens were secured by inserting the wooden board into the holding jig of the material testing machine (Instron 4467; Instron, Norwood, MA, USA). The compression jig was then adjusted so that the force was applied to the highest point of the lateral condyle. Compression was performed in the proximal direction of the bone at a speed of 10 mm/s and continued until failure occurred. Failure was defined as implant breakage or bending, 5 mm of displacement, complete fracture of the humeral condyle, or a sudden drop in the load–displacement curve.

### 2.4. Statistical Analysis

Statistical analyses were conducted using SPSS software (Version 29.0.2.0; SPSS Inc., IBM, Chicago, IL, USA). Given that each of the three groups comprised fewer than ten participants, the Kruskal–Wallis test was initially performed. This was subsequently followed by post hoc Mann–Whitney U tests. To ensure a 95% confidence level across the three methods, the Bonferroni correction was applied. Consequently, statistically significant difference was considered at a *p*-value below 0.017 in the Mann–Whitney U tests.

## 3. Results

### 3.1. Maximum Failure Loads

**Figure 5:**
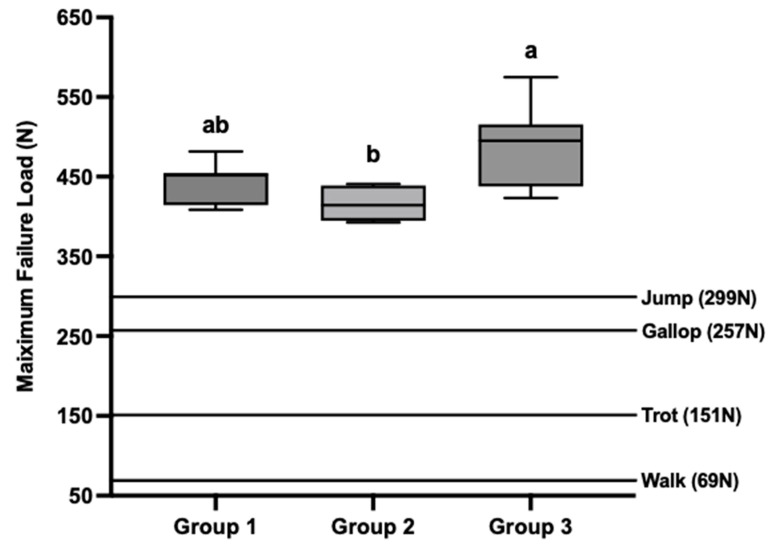
Box–Whisker plot of loads for each group at maximum failure load and the load values based on previous studies. Statistically different groups were denoted by distinct letters above the box plot (*p* < 0.017). Groups sharing the same letter were not significantly different. The maximum failure load was significantly higher in Group 3 compared to Group 2 (*p* < 0.017). However, there were no significant differences in maximum failure load between Group 1 and Group 2 or between Group 1 and Group 3 (*p* > 0.017). The lines on the graph showed the load to the forelimb in walking, trotting, galloping [12,13,14] and jumping [15]. Each load was calculated in an average weight of 10.99 kg for the cadavers used in this study. Group 1: 3.0 mm cortical screw with lag fashion. Group 2: 3.0 mm PTCS. Group 3: 3.5 mm FTHCS.

The mean maximum failure loads were 441.29 ± 29.78 N, 413.39 ± 20.36 N, and 488.35 ± 50.57 N for Groups 1, 2, and 3, respectively (Table 1). The mean maximum failure load was highest in Group 3 (Figure 5). Statistical analysis revealed a significant difference in maximum failure load between Groups 2 and 3 (*p* = 0.014). However, there were no significant differences between Groups 1 and 2 or between Groups 1 and 3 (*p* > 0.017) (Figure 5).

### 3.2. Types of Failure

**Figure 6:**
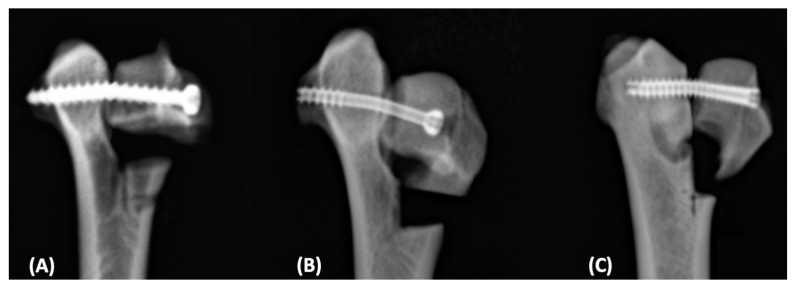
Radiographic images showing failure after the axial compression test. Failure mode of samples was fixed using different screw types, as illustrated above: (**A**) Group 1, a 3.0 mm self-tapping cortical screw inserted in a lag fashion was bent. (**B**) Group 2, a 3.0 mm PTCS was bent. (**C**) Group 3, a 3.5 mm FTHCS was bent. Group 1: 3.0 mm cortical screw with lag fashion. Group 2: 3.0 mm PTCS. Group 3: 3.5 mm FTHCS.

Types of failure were recorded after each experiment (Figure 6). In Group 1 (cortical screw), screw bending was the most common failure type, occurring in all seven samples (*n* = 7). Additionally, one sample (*n* = 1) exhibited a medial condyle fracture concurrently with screw bending. In Group 2 (PTCS), screw bending was observed in all seven samples (*n* = 7). Similarly, in Group 3 (FTHCS), screw bending was the predominant failure type, occurring in all seven samples (*n* = 7), with one sample (*n* = 1) also experiencing a medial condyle fracture, as seen in Group 1 (Table 1).

## 4. Discussion

In this study, a comparative analysis was conducted to evaluate the biomechanical properties of self-compressing screws and cortical screws inserted using the lag fashion technique in cadaveric models of humeral condylar fractures.

Two distinct types of self-compressing screws were examined: the PTCS and the FTHCS, each utilizing a unique fixation method. The PTCS facilitates interfragmentary compression by allowing its threaded section to engage with bone beyond the fracture site. As the screw head is pressed against the near cortex, compression across the fracture site is generated, effectively functioning as a lag screw without the necessity of a gliding hole [7]. On the other hand, the FTHCS was selected for its ability to be inserted rapidly, similar to the PTCS, and its capacity to prevent complications related to screw head movement, such as irritation of the surrounding soft tissues. Additionally, this screw type ensures adequate biomechanical strength essential for stabilizing fractures in human patients [9,16,17] and has recently been evaluated in cadaveric models of femoral neck fractures in dogs [8].

The proposed hypothesis posited that the biomechanical stability of the PTCS and the FTHCS would be similar to those of the cortical screw inserted using the lag fashion technique when subjected to a single axial compression test. The results of this study revealed that the maximum failure loads of cortical screws inserted using the lag technique did not differ significantly from those of the PTCS and the FTHCS. These findings suggest that both the PTCS and the FTHCS may serve as effective alternatives to traditional cortical screw methods for the fixation of humeral condylar fractures, a conclusion that aligns with previous research comparing cortical screws and partially threaded headless screws [6].

However, it was observed that the maximum failure loads of FTHCS were higher than those of PTCS. This discrepancy may be attributed to differences in screw diameters, as both types share the same cannulation diameter. Statistically, no significant difference was found between the cortical screw inserted using the lag fashion technique and the FTHCS. In contrast, the PTCS exhibited a significant difference compared to the FTHCS. This difference is potentially due to the presence or absence of cannulation in screws of identical diameters.

The standard deviation of Group 3 was approximately 10% of the mean value, while the other groups showed about 5%. In our opinion, the difference between the standard deviations might come from the presence of a head. As mentioned, the fully threaded headless screw does not provide compression with the screw head [8]. Therefore, as the locations of the narrowest parts of the condyles were different in each sample, the difference in fracture-line location was set, and this might affect compressive variation. We adjusted screws to be engaged properly by matching the far bone fragment with thread length in the PTCS or the pilot hole length in the lag screw.

The maximum failure loads in this study differed from those reported in previous research. Rochereau et al. (2012) reported ultimate failure loads for a 4.0 mm PTCS and a 4.0 mm cancellous screw as 1078 ± 231 N and 1261 ± 261 N, respectively. The body weights of the cadavers ranged from 35 to 45 kg, with a median of 40 kg. In that study, the load was applied in a single ramp at a constant displacement speed of 1 mm/s [18]. M.N. Gonsalves et al. (2016) reported failure loads of 1222 ± 320 N for 3.5 mm cortical screws and 979 ± 209 N for 3.0 mm partially threaded headless screws. The mean body weight of the cadavers was 30.9 ± 5.3 kg. In their study, the load was applied at a rate of 100 N/s in a single ramp to failure [6].

The displacement at the maximum failure load point was comparable to that observed in our experiments. Since our study also confirmed the failure mode of each sample via radiographs—demonstrating that all implants received proper compression forces—the differences in maximum failure loads may result from variations in compression speed, the mean body weight of the cadavers, and the implants used.

The peak vertical force exerted by a dog’s forelimb has been reported to reach 64%, 140%, 238%, and 278% of the body weight during activities in walking, trotting, galloping, and jumping, respectively [12,13,14,15]. Considering the average body weight in this study was 10.99 kg, the estimated peak vertical forces on the forelimbs during walking, trotting, galloping, and jumping were calculated to be 69 N, 151 N, 257 N, and 299 N, respectively. When compared to the maximum failure loads reported for each screw group (Figure 5), these results indicate that all screw groups demonstrated sufficient stability to withstand the forces experienced during these activities.

Both cortical screws inserted using the lag technique and PTCS are capable of achieving the necessary compression for fracture stabilization [18]. However, the protrusion of the screw head may lead to irritation of the surrounding soft tissues. Previous studies have reported seroma formation rates ranging from 3.99% to 31.6% following surgical management of humeral condylar fractures [19,20]. In contrast, FTHCs offer the advantage of rapid insertion and compression independent of thread positioning. After fixation, these screws are fully embedded within the bone, thereby preventing any protrusion above the cortex. This characteristic minimizes the risk of soft tissue irritation and potentially reduces the incidence of seroma formation, as reported in human medicine [20].

Additionally, less invasive surgical approaches to humeral unicondylar humeral condylar fracture with PTCS minimize the need for significant muscle and joint capsule dissection, which has recently been performed in clinics [21]. Both cannulated screws used in this study are expected to be utilized in minimally invasive surgical approaches for humeral condylar fractures [21].

PTCSs generate compression by engaging the threaded section with the bone beyond the fracture site. Consequently, smaller fragments of the far cortex may result in incomplete thread engagement, potentially compromising compression [8]. On the other hand, FTHCSs provide compression irrespective of the positioning of the threaded portion. However, if the adjacent fragment is too thin, it may prevent sufficient compression from being achieved [22]. Therefore, selecting the appropriate screw type appears to be critically dependent on the specific location and characteristics of the fracture.

While some reports suggest that interfragmentary compression may be lower with PTCSs compared to traditional cortical screws [23], other studies have indicated that PTCSs offer comparable pull-out strength to that of non-cannulated screws [24]. Anatomical reduction was successfully achieved in all cases within this study, as confirmed through both visual inspection and radiographic imaging, consistent with findings from previous studies [18]. Additionally, as reported by Bulut et al. (2019), FTHCSs provided sufficient compression for anatomic reduction, similar to the performance of PTCSs observed in this study [17].

During the compression test, all sample failures were characterized by proximal displacement of the lateral fracture fragment, accompanied by separation at the distal part of the fracture line. In Groups 1 and 3, each sample exhibited a medial condyle fracture that was distally parallel to the long axis of the screws, along with simultaneous implant bending. These results appear to be attributable to the fulcrum effect induced by the osteotomy of the lateral portion for experimental purposes of compression and may be unlikely to occur with a stable lateral supracondylar crest.

There are several limitations to this study. First, the body weights of cadavers were limited to those weighing approximately 11 kg. Further research is required to evaluate the reliability of these screw types in both small and large breed dogs. Second, as we removed all soft tissues of the cadaveric humeri, the muscle forces were excluded, which could potentially alter the load conditions in actual physiological motion. Plus, clinically relevant cyclic loading was not examined, so this study was unable to accurately simulate the natural motion of the elbow. Finally, the limited number of specimens may have influenced the outcomes, underscoring the necessity for further studies with larger sample sizes in each group. However, this study is meaningful in that it compared screws of the same manufacturer and identical material in cadaveric models and showed the potential for clinical use of self-compression screws.

## 5. Conclusions

In conclusion, partially threaded cannulated screws and fully threaded headless cannulated screws demonstrated enough fixation strength compared to the conventional cortical screw with lag technique. This suggests that these two methods could be alternatives for repairing humeral condylar fractures in dogs.

## Figures and Tables

**Table 1:** Maximum failure load of lateral humeral condyle compression and types of failure for each group at maximal failure load. The maximum failure load significantly differed between Groups 2 and 3 (*p* < 0.017). However, there was no statistically significant difference in the maximum failure load between Groups 1 and 2 or between Groups 1 and 3 (*p* > 0.017). Types of failure were recorded after each experiment. In Group 1 and Group 3, screw bending was the most common failure type, occurring in all seven samples (*n* = 7). Additionally, one sample in each of these groups (*n* = 1) exhibited a medial condyle fracture concurrently with screw bending. In Group 2, only screw bending was observed in all seven samples (*n* = 7).

Groups	Maximum Failure Load (N, Mean ± SD)	Failure Types	Frequency
Group 1 (*n* = 7)	441.29 ± 29.78 N	Screw bending	7
		Medial condyle fracture	1
Group 2 (*n* = 7)	413.39 ± 20.36 N	Screw bending	7
Group 3 (*n* = 7)	488.35 ± 50.57 N	Screw bending	7
		Medial condyle fracture	1

N: Newton; SD: standard deviation. Group 1: 3.0 mm cortical screw with lag fashion. Group 2: 3.0 mm PTCS. Group 3: 3.5 mm FTHCS.

## Data Availability

The data presented are available within this study.
